# Can YOLO Detect Retinal Pathologies? A Step Towards Automated OCT Analysis

**DOI:** 10.3390/diagnostics15141823

**Published:** 2025-07-19

**Authors:** Adriana-Ioana Ardelean, Eugen-Richard Ardelean, Anca Marginean

**Affiliations:** Computer Science Department, Technical University of Cluj-Napoca, 400114 Cluj-Napoca, Romania; ardelean.sz.adriana@student.utcluj.ro (A.-I.A.); anca.marginean@cs.utcluj.ro (A.M.)

**Keywords:** optical coherence tomography, YOLO, object detection, neural networks, retinal OCT

## Abstract

**Background:** Optical Coherence Tomography has become a common imaging technique that enables a non-invasive and detailed visualization of the retina and allows for the identification of various diseases. Through the advancement of technology, the volume and complexity of OCT data have rendered manual analysis infeasible, creating the need for automated means of detection. **Methods:** This study investigates the ability of state-of-the-art object detection models, including the latest YOLO versions (from v8 to v12), YOLO-World, YOLOE, and RT-DETR, to accurately detect pathological biomarkers in two retinal OCT datasets. The AROI dataset focuses on fluid detection in Age-related Macular Degeneration, while the OCT5k dataset contains a wide range of retinal pathologies. **Results:** The experiments performed show that YOLOv12 offers the best balance between detection accuracy and computational efficiency, while YOLOE manages to consistently outperform all other models across both datasets and most classes, particularly in detecting pathologies that cover a smaller area. **Conclusions:** This work provides a comprehensive benchmark of the capabilities of state-of-the-art object detection for medical applications, specifically for identifying retinal pathologies from OCT scans, offering insights and a starting point for the development of future automated solutions for analysis in a clinical setting.

## 1. Introduction

Optical Coherence Tomography (OCT) [1] is a non-invasive technique used for cross-sectional tissue imaging in three dimensions, which has been extensively used in ophthalmology as it provides high-resolution and detailed images of the retina. OCTs have been used for detecting and tracking the progression of eye diseases (such as Age-related Macular Degeneration and Diabetic Macular Edema), as well as monitoring the effectiveness of treatments. It has become valuable for the analysis of the human eye due to its non-invasive nature, as diagnosis through biopsy is not available. OCTs offer fast scanning rates, allowing for real-time visualization and offer a higher resolution [2,3] than ultrasounds or magnetic resonance imaging.

OCT has similar working principles to ultrasound, as both apply waves to the tissue under examination, which echo off the tissue structure [1]. As the waves return, their delay is used to measure the depth. In contrast to ultrasound, OCT uses near-infrared light, which travels much faster. Due to the increasing volume of OCT scans in clinical practice, manual analysis is no longer a feasible option, indicating a new venue of research as the automated detection of OCT scans for the identification of biomarkers and pathology.

Manual annotation and analysis of OCT images present challenges as the process itself is extremely time-consuming [4], requiring significant time to identify and delineate pathological regions. As such, the growing volume of data may reach a point where it exceeds the available time of experts to analyze. Moreover, inter-expert variability can lead to inconsistent diagnoses, especially for early-stage diseases where opinions may vary. Thus, the use of neural networks [5,6,7,8] for automated detection can greatly help and improve the field of retinal disease detection with the purpose of early identification and prevention.

Recent advances in deep learning, specifically in object detection, show a heightened potential for addressing these challenges. Among the vast expanse of approaches, the “You Only Look Once” (YOLO) [9] family of models has perhaps received the most attention, with many variations and versions having been developed in the last few years. This can be attributed to the balance they provide between speed and accuracy, which is a requirement for real-time clinical analysis.

YOLO has been shown to have various applications in medical tasks. It has been shown to have a high performance in coronary OCT, outperforming other architectures such as a Single Shot Multibox Detector (SSD) [10]. YOLOv8 and YOLOv9 have been analyzed for the task of feature detection for diabetic retinopathy from retinal fundus images [11], showing limitations for the detection of microaneurysms but highlighting the progress achieved in this field. The YOLOv7 model has been modified and used in automated blood vessel detection in pathological images [12], outperforming other traditional algorithms. In a systematic literature review [13], the YOLO models were identified as the most frequently applied and the most powerful due to several factors: high accuracy, low computational cost, simple architectures, and robustness. This review [13] also indicates the wide range of medical applications of the YOLO versions: brain tumor detection, coronary artery segmentation, pulmonary embolism detection, kidney disease localization, skin lesion detection, and glaucoma detection. Recent work has also shown that YOLO architectures are not always the best performing, especially for the detection of microaneurysms in diabetic retinopathy [14] on OCT scans. Not only object detection was used for OCT scans, but also image segmentation. In a recent study [5], it was found that several architectures, such as Feature Pyramid Networks, Unet, and Unet++, using a Resnext backbone are able to perform image segmentation of retinal layers and fluids in OCT scans for the identification of biomarkers.

These previous studies have explored earlier YOLO versions in medical analysis. The recently introduced YOLOv12 integrates an attention-centric design aligned to today’s most explored deep learning approaches, transformers. This model represents a potential advancement in retinal OCT analysis, which has not yet been able to be evaluated. Existing comparative studies often focus their analysis on a single dataset or a limited number of models, lacking a comprehensive benchmark across the state of the art. This study attempts to address these gaps by providing a comprehensive evaluation of retinal pathology detection in OCT images.

In this work, we evaluate several modern object detection models in an attempt to find the current status of detection performance for retinal OCT. Our contributions include: (1) the performance evaluation of the latest YOLO versions (from YOLOv8–v12) and alternative architectures (RT-DETR, YOLO-World, YOLOE) across two diverse datasets for object detection on OCT scans, (2) an analysis of performance trade-offs between accuracy, speed, and computational efficiency in the clinical context of the data, and (3) insights for the deployment of automated OCT analysis systems in clinical settings.

## 2. Materials and Methods

This study employs a comparative analysis to evaluate state-of-the-art object detection models for retinal pathology identification in OCT images. We systematically compared traditional CNN-based architectures (YOLOv8–v11) with newer attention-based and transformer-based approaches (YOLOv12, RT-DETR, YOLO-World, YOLOE) across two datasets representing different pathological complexity levels. Two complementary datasets were used in this work: the AROI dataset [15], which focuses specifically on fluid detection in AMD cases, and the OCT5k dataset [16], which provides broader pathology coverage including drusen, geographic atrophy, and various retinal abnormalities. Recent studies [7,8,17] have evaluated the ability of various image segmentation models on the OCT scans dataset, even on the AROI dataset. However, the application and benchmarking of object detection models in this domain remains lacking; thus, in this work, we attempt to fill this gap.

The experimental design contains standardized training parametrization for a fair comparison, including hyperparameter optimization and data augmentation. The performance of the models was analyzed across multiple metrics and dimensions, including detection accuracy (mAP@50, mAP@50-95, and others) and computational efficiency (inference time, FLOPs).

### 2.1. YOLO

The “You Only Look Once” (YOLO) [9] object detection algorithm was initially introduced in 2015. The YOLO architecture managed to revolutionize real-time object detection by unifying region proposal and classification within a single neural network. This approach divided the image into a grid, directly predicting bounding boxes and class probabilities for each cell, which significantly reduced computation time and enabled end-to-end learning. YOLOv1 utilized a simplified CNN backbone and was good at accelerating the detection process. However, it faced challenges with small objects and spatial constraints, with each grid cell only predicting two boxes and having limited context.

The YOLO series has since undergone a decade of continuous evolution [18], progressing through numerous versions starting from YOLOv2, and culminating in the recently unveiled YOLOv12. Each iteration introduced incremental technological advancements aimed at enhancing speed, detection accuracy, and computational efficiency. Beginning with YOLOv2 [18,19,20], this version was built upon YOLOv1 by improving the resolution at which the model operated by incorporating techniques like batch normalization and anchor boxes from Faster R-CNN to improve training and performance. YOLOv3 [18,19,21] further advanced detection capabilities by implementing multi-scale predictions through Feature Pyramid Networks (FPNs) and a deeper network architecture, Darknet-53, which specifically allowed for better detection of smaller objects. YOLOv4 [18,19,22] marked a significant point by integrating advanced features like CSPDarknet-53 and PANet in its architecture with Mish activation, alongside new data augmentation techniques like Mosaic and CutMix, setting new standards for speed and accuracy. Subsequently, YOLOv5 introduced by Ultralytics [18,19,23] (version 8.3.167), focused on ease of use and performance, providing a streamlined PyTorch-based framework (version >=1.8.0) with improved backbone, neck, and head designs, and it also featured an auto-anchor learning that adjusted the sizes of anchors during training. YOLOv6 [18,19,24] introduced a novel backbone to the YOLO architectures, EfficientRep with RepOptimizer, focusing on efficiency. YOLOv7 [18,19,25] brought in a trainable bag of freebies and the Extended Efficient Layer Aggregation Network (E-ELAN), which achieved increased efficiency. YOLOv8 [18,19,23], also from Ultralytics [23], refined the architecture with the C2f module and transitioned to an anchor-free detection, allowing for more specialized and simpler detection. YOLOv9 [18,19,26] addressed information degradation in deep networks by introducing the Generalized Efficient Layer Aggregation Network (GELAN) architecture and Programmable Gradient Information (PGI). YOLOv10 [18,19,27] achieved remarkable real-time efficiency by eliminating non-maximum suppression (NMS) and implementing a dual assignment strategy, along with architectural innovations like lightweight classification heads. The latest before YOLOv12, YOLOv11 [18,19,23], integrated the C3k2 block, SPPF, and C2PSA (Convolutional Block with Parallel Spatial Attention) for enhanced feature extraction and spatial attention, balancing precision and efficiency for diverse applications.

### 2.2. YOLOv12

YOLOv12 [18,19,23,28], introduced in February 2025, although based on the YOLO framework, has made a significant shift in approach from its predecessors, moving towards an attention-centric design, achieving state-of-the-art performance in both accuracy and efficiency while maintaining real-time detection capabilities. Unlike previous YOLO methods that primarily relied on CNN-based improvements for speed and accuracy, YOLOv12 integrates attention mechanisms while maintaining the high inference speed for real-time object detection [19,28] and, thus, challenges the traditional view that attention-based models are too slow for real-time. YOLOv12 has managed to achieve this through innovations such as the Area Attention module, which reduces computational complexity and maintains a large receptive field, and Residual Efficient Layer Aggregation Networks (R-ELAN), designed to improve feature aggregation and address optimization challenges, particularly in larger models. Additional architectural refinements include using FlashAttention to handle memory access issues, adjusting the MLP ratio, implementing attention with convolutions for efficiency, removing positional encoding, and incorporating a large separable convolution (Position Perceiver) to aid positional perception.

The development of YOLOv12 involved extensive analyses [28] to validate its design and performance. Comparative studies demonstrate that YOLOv12 achieves state-of-the-art results in accuracy (mAP) with superior speed (latency) and reduced computational cost (FLOPs) compared to popular methods like YOLOv6, YOLOv8, YOLOv10, YOLOv11, and RT-DETR variants across different model scales. Ablation studies specifically confirmed the effectiveness of R-ELAN (showing its necessity for stable training in larger models) and Area Attention (demonstrating significant speedups). Diagnostic studies also evaluated various architectural choices, including attention implementation methods, the hierarchical design, training epochs, the Position Perceiver, positional embedding, MLP ratio, and the impact of FlashAttention. Moreover, visualizations through heatmaps show YOLOv12’s improved object perception compared to earlier versions. The YOLOv12 model uses a composite loss function for training, which is composed of box loss (with a factor of 7.5), class loss (with a factor of 0.5), and a distribution focal loss (with a factor of 1.5). The box loss quantifies how accurately do the predicted bounding boxes match the ground truth bounding boxes, the class loss evaluates the model’s ability to classify the objects within the predicted bounding boxes, and the distribution focal loss is a loss function designed to improve object detection accuracy by focusing on hard to guess examples by predicting the distribution of possible box offsets instead of the exact coordinates.

### 2.3. Other Object Detection Models

Real-Time Detection Transformer (RT-DETR) [29,30,31] provides an alternative approach that removes the need for non-maximum suppression through the use of an end-to-end transformer. RT-DETR is a redesigned version of DETRs with a focus on increasing computational efficiency while maintaining/increasing accuracy. The traditional transformer encoder is substituted for a hybrid encoder, which allows for increased speed by separately processing the intra-scale interaction and cross-scale fusion of features. The increase in detection accuracy is achieved through a query selection mechanism. RT-DETRv2 [31] improves upon its predecessor through an improved training strategy, specifically dynamic data augmentation and scale-adaptive hyperparameters. It improves multi-scale detection through the introduction of Selective Multi-Scale Sampling.

YOLO-World [32] is an extension of the YOLO approach based on YOLOv8 [11] with open-vocabulary detection capabilities through vision-language modeling. Its incorporation of vision-language modeling demonstrated an increased capacity for identifying a broad array of objects in zero-shot scenarios with high efficiency. YOLO-World introduces a Re-parameterizable Vision-Language Path Aggregation Network (RepVL-PAN), which allows interaction between visual and linguistic information and region-text contrastive loss, aligning visual regions with textual descriptions. It demonstrated an increased ability to detect objects, making it suitable for real-world applications. YOLOE [33] is another extension of the YOLO approach based on YOLOv8 [11] and YOLOv11 [11] that was designed for object detection and instance segmentation of images guided through prompt mechanisms. YOLOE supports text prompts through its Re-parameterizable Region-Text Alignment (RepRTA) strategy, it also supports visual prompts through Semantic Activated Visual Prompt Encoder (SAVPE), and it allows for prompt-free scenarios through a Lazy Region-Prompt Contrast (LRPC) strategy. Its ability to integrate contextual information through its prompt-guided approach might be beneficial as the identification of context-relevant information is one of the most important preprocessing steps [34]. For its object detection functionality, the YOLOE model also has a composite loss, which includes a box loss, a class loss, and a distributed focal loss.

### 2.4. Performance Evaluation

Although it is a common practice, the assessment of performance through a single performance metric may be perilous [35,36] if the metric does not cover all relevant aspects, leading to a misleading and biased assessment.

Precision refers to the percentage positive predictions that are actually correct and can be computed as the ratio of true positives (TP) (correct detection of a class when compared to the ground truth) to the sum of true positives and false positives (FP) (incorrect detection of a class as nothing actually exists when compared to the ground truth). Precision evaluates the ability to avoid false alarms (false positives) and can be computed through the following formula:

$$Precision=\frac{TP}{TP+FP}$$

Recall refers to the percentage of actual positives that are correctly identified and can be computed as the ratio of true positives to the sum of true positives and false negatives (FN) (incorrect detection of a class as nothing was detected when compared to the ground truth). Recall evaluates the ability to avoid missing detections (false negatives) and can be computed through the following formula:

$$Recall=\frac{TP}{TP+FN}$$

*F1* score refers to the harmonic mean of precision and recall, which is a balanced measure of performance. As it uses both precision and recall, it accounts for both false positives and false negatives, and it is especially relevant when both precision and recall are important. It can be computed through the following formula:

$$F1=\frac{2*Precision*Recall}{\left(Precision+Recall\right)}$$

Intersection over Union (IoU) refers to the overlap between predicted and true bounding boxes and is computed as the ratio between the intersection area and the union area of two bounding boxes. IoU evaluates the ability to delineate object boundaries rather than simply detection and can be computed through the following formulae:

$$IoU=\frac{\mathrm{Intersection}\;\mathrm{area}}{\mathrm{Union}\;\mathrm{area}}$$

Mean Average Precision at an IoU threshold of 0.5 (mAP@50) represents the Average Precision (AP) (area under the precision–recall curve) computed for each class when detections are correct and they reached an IoU of at least 0.5 with true bounding boxes.

$$mAP@50=\frac{1}{\left|C\right|}\sum_{c\in C}{AP}_{c}(IoU\geq 0.5)$$

where *C* represents the set of classes and c represents a single class of *C*.

Mean Average Precision at IoU thresholds from 0.5 to 0.95 (mAP@50-95) represents the AP scores across multiple IoU thresholds from 0.5 to 0.95 with an increment of 0.05, and it captures the ability to localize across varying levels of requirements, thus penalizing detections that are only marginally aligned with true objects. mAP@50-95 can be computed through the following formula:

$$mAP@50\text{-}95=\frac{1}{\left|C\right|}\sum_{c\in C}\left(\frac{1}{\left|T\right|}\sum_{t\in T}{AP}_{c}(IoU\geq t)\right)$$

where *C* represents the set of classes and *c* represents a single class of *C*, and T represents the set of thresholds {0.5, 0.55, …, 0.95}.

### 2.5. Data

OCT imaging is increasingly used in ophthalmic clinical practice [15]. The growing number of available OCT images highlights the importance of robust algorithms for automatic segmentation [15]. Automatic segmentation is crucial because manual segmentation is time-consuming and infeasible in clinical practice, while segmentation of retinal structures and pathological biomarkers is necessary for more accurate diagnosis and effective therapy [15].

#### 2.5.1. AROI Dataset

The Annotated Retinal OCT Images (AROI) dataset [15] is described as a step towards developing a robust artificial intelligence system in ophthalmology. The absence of publicly available databases is a major issue that hinders the validation of algorithms and may lead to reproducibility/replication issues. The AROI dataset was developed specifically to address these issues in the field of ophthalmology, particularly for OCT images.

**Figure 1:**
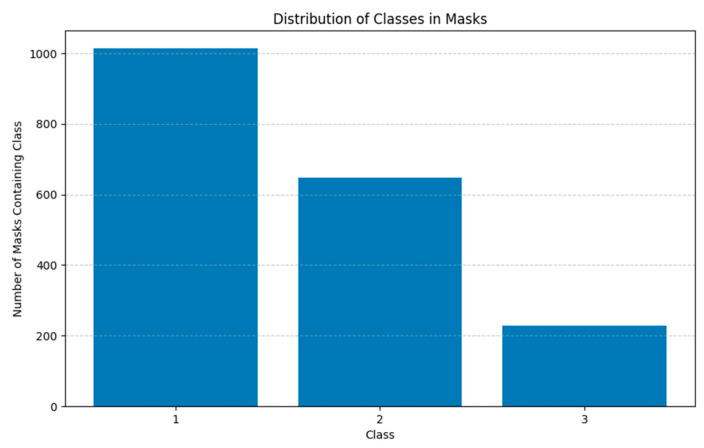
Class distribution of biomarkers in the AROI dataset (class 1 represents PED, class 2 represents SRF, and class 3 represents IRF).

The AROI dataset [15] consists of 1136 annotated B-scans from 24 patients suffering from neovascular Age-related Macular Degeneration (nAMD). For each annotated B-scan, images are prepared for semantic segmentation through manual annotation by an ophthalmologist, with eight classes representing areas above and below the retina, areas between annotated layer boundaries, and the three retinal fluids. The annotated fluids are pigment epithelial detachment (PED), subretinal fluid/subretinal hyperreflective material (SRF), and intraretinal fluid (IRF). These annotations provide the ground truth necessary for training and validating machine learning algorithms for automatic segmentation, and their distribution is presented in Figure 1.

**Figure 2:**
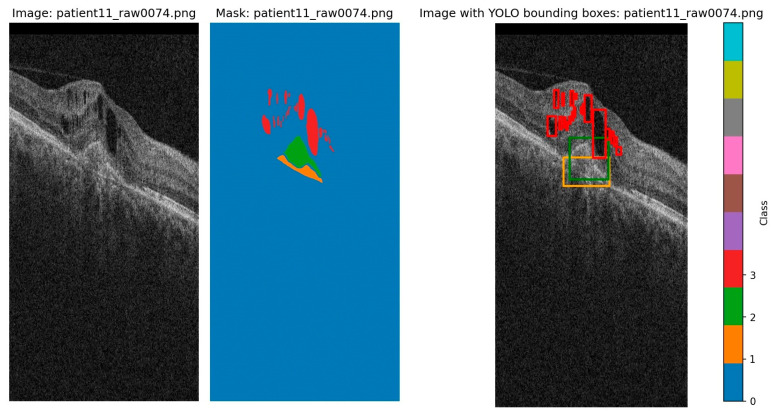
Original image, original segmentation, and YOLO labels transform (class 1/orange represents PED, class 2/green represents SRF, and class 3/red represents IRF). There tend to be more instances of IRF that cover small regions, which may even overlap when translated to bounding boxes. Due to the segmentation, even the bounding boxes of PED and SRF overlap with each other, and sometimes with the smaller IRF.

The annotations are in a mask format for image segmentation and were converted to bounding boxes for this project. Bounding boxes were determined as the minimum and maximum for each axis for each blob of each class and were then converted to the yolo format. The original segmentation and the transformation to YOLO bounding boxes are shown in Figure 2.

#### 2.5.2. OCT5k Dataset

The OCT5k dataset [16] is described as a comprehensive collection of retinal OCT images. Previous publicly available open access OCT datasets for retinal layer segmentation have been limited in scope, often being small in size. OCT5k provides pixel-wise manual labels for 1672 OCT scans featuring three different disease classes (Age-related Macular Degeneration, Diabetic Macular Edema, and healthy), in contrast to previous datasets, which were specific to a single disease. The wider set of diseases supports the development of more sophisticated algorithms required for better generalization across varying pathological conditions. These scans were also graded by multiple people in order to reduce bias [16], which is another improvement over previous datasets, which often contained a single grading.

**Figure 3:**
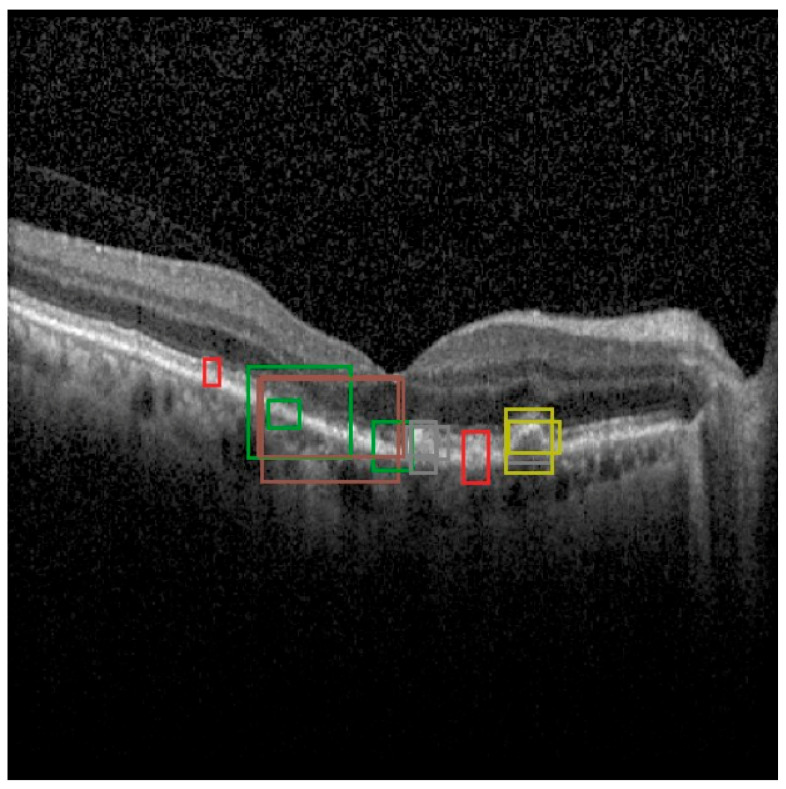
Example of an image from the OCT5k dataset with the bounding boxes given for each class instance (class 1/orange represents Choroidalfolds, class 2/green represents Fluid, class 3/red represents Geographicatrophy, class 4/purple represents Harddrusen, class 5/brown represents Hyperfluorescentspots, class 6/pink represents PRlayerdisruption, class 7/gray represents Reticulardrusen, class 8/olive represents Softdrusen, class 9/cyan represents SoftdrusenPED).

Moreover, out of 1672 scans, OCT5k provides 4698 bounding box annotations for a subset of 566 scans across nine classes of disease biomarkers [16]. These classes include entities such as soft drusen, hard drusen, photoreceptor layer disruption, retinal fluid, geographic atrophy, and more [16]. The bounding boxes provided offer an alley of analysis for object detection approaches such as the YOLO architecture. OCT5k provides 566 images with bounding boxes that have been converted to the yolo format. An example of an OCT5k image and its bounding boxes is shown in Figure 3.

## 3. Results

The latest versions of YOLO (v8 to v12) have been compared against each other on the AROI dataset in a first analysis. The training procedure has been performed on the same random subset of 70% of the original dataset, while the validation and testing subsets contained 20% and 10%, respectively. They were trained for 50 epochs across the entire training set (with images that have been rescaled to 640 × 640) with batches of 16 using the AdamW optimization with a starting learning rate of 1 × 10^−4^, which will be reduced to 1 × 10^−6^ during the training process. A small penalty to large weights is added through a weight decay of 5 × 10^−4^. They used a training learning rate warmup of three epochs, which stabilizes the training as the initial learning is reached through a gradual increase.

To reduce the possibility of overfitting the models even further, data augmentation was also applied to increase the data diversity. The training images in the HSV format were randomly shifted as hue = 0.015, saturation = 0.7, and value = 0.4, allowing the model to be invariant to lighting conditions. The images were also augmented through random rotations of up to ±10 degrees, translations of up to 10% on either axis, and random zoom-ins/zoom-outs of up to 20%. Mixup augmentations were used in the training process, which combine two images into a single composite one, increasing generalizability by introducing noise and variability. While mosaic augmentations create combinations of multiple images to simulate high complexity conditions. Mosaic augmentations were disabled for the last 10 epochs to allow the model to stabilize. Through a random probability, each image may have been modified to include a subset of the augmentations mentioned above.

The AROI dataset [15] was created with a focus on AMD fluids, while OCT5k covers a wider spectrum of pathologies; thus, we report YOLO’s performance separately on each dataset. All experiments were conducted on the AROI fluid class annotations (where the task is multi-class fluid detection) and on OCT5k (where all classes of pathological markers were kept).

### 3.1. Performance Analysis of YOLO Versions on the AROI Dataset

In Table 1, we present the results on the testing dataset of the YOLOv8 to YOLOv12 versions, RT-DETR, YOLO-World, and YOLOE run with a confidence threshold of 0.25 (confirmed by the F1-confidence curves) and an IoU threshold of 0.5 (found empirically to offer the best results across the chosen metrics). Across the traditional YOLO family of models, YOLOv12 achieved the highest overall performance with a mAP@50 of 0.712, representing a 2.8% improvement over YOLOv11, its predecessor, and an improvement of 7.1% over YOLOv9, the lowest-performing model of the YOLO family. Moreover, the higher performance of YOLOv12 from the perspective of mAP@50-95 indicates better localization precision across varying IoU thresholds than the other models. When considering computational efficiency, YOLOv12 maintains a low inference time of just 4.9 ms and the lowest computational cost of all models of just 21.2 GFLOPs. This indicates that YOLOv12 is one of the most adequate models when a trade-off between accuracy and efficiency is required.

Despite its newer transformer-based approach and its results on other domains [14], RT-DETR underperformed when compared to any other YOLO-based approach, as it obtained a mAP@50 of only 0.566, significantly lower than the other models. Moreover, it has the highest inference time of 15.3 ms, being more than two times slower than any of the other models, and it incurs the highest computational cost of 103.4 GFLOPs.

YOLOE demonstrated the best performance overall with a mAP@50 of 0.725, likely due to its prompt-guided detection mechanisms that may be particularly suited for medical imaging tasks where contextual information is key, and the lowest inference time of only 4.1 ms. Its only disadvantage when compared to the second-best-performing model, YOLOv12, is its higher computational cost (YOLOE requires 35.3 GFLOPs vs. YOLOv12 requires 21.2 GFLOPS.

The results of YOLOv12 on the test partition of the AROI dataset are presented in more detail in Table 2. The highest performance of YOLOv12 was achieved on the SRF class with a mAP@50 of 0.834 and a recall of 0.738, suggesting a high sensitivity for this particular biomarker. For the PED biomarker, the results are also promising with a mAP@50 of 0.749 and balanced precision–recall characteristics (0.786 precision, 0.631 recall). However, the most problematic class was the IRF; the model only managed to achieve a mAP@50 of 0.553 and a significantly lower recall (of only 0.41) when compared to the other classes.

**Figure 4:**
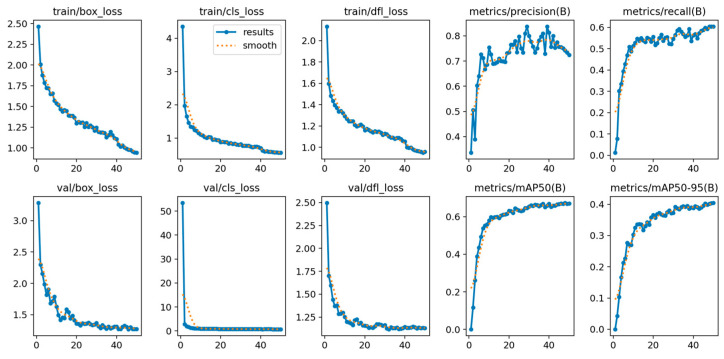
YOLOv12 train and validation on the AROI dataset showing the model is capable of attaining stability during the training process with minimal overfitting due to the alignment of training and validation.

YOLOv12’s training is shown in Figure 4, showing its convergence. The model achieved a stable training with minimal overfitting, evidenced by the alignment between training and validation metrics during the training process. The loss curves show a continuous reduction, while the metrics show steady improvement across epochs.

**Figure 5:**
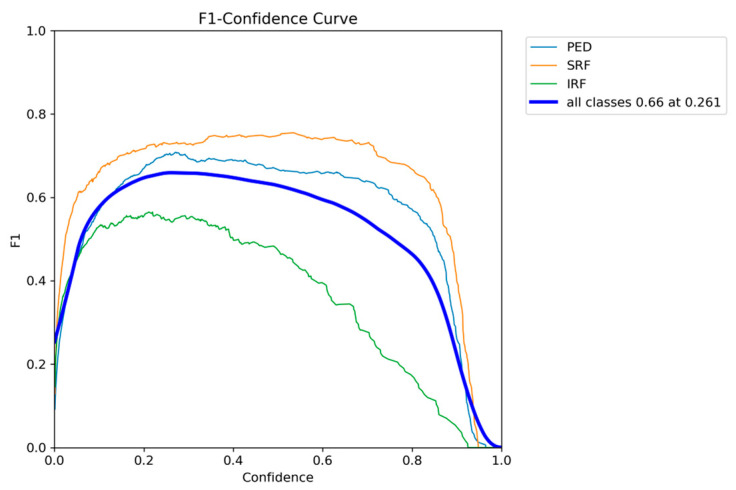
YOLOv12 F1-confidence curve on the AROI dataset showing the severe drop in performance as the confidence threshold increases for the IRF class.

The F1-confidence curve presented in Figure 5 shows that optimal performance can be achieved at a confidence threshold of around 0.3, with the F1 score of all classes having the highest performance of 0.66 at a threshold of 0.261. By inspecting the individual class curves, SRF and PED maintain their F1 scores on a broad range of confidence thresholds; however, IRF drops rapidly as the threshold value increases.

**Figure 6:**
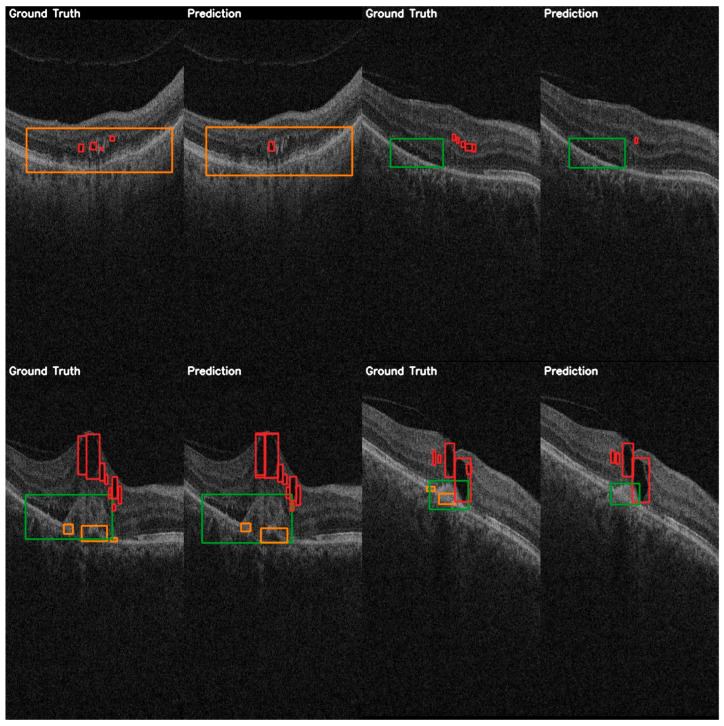
YOLOv12 validation examples on the AROI dataset showing the general capability of the model to correctly identify the PED (orange) and SRF (green), while the IRF (red) class is harder to distinguish from the background, and aligning the bounding box is harder to accomplish due to it occupying only small regions.

For a more practical performance analysis, the validation examples can be viewed in Figure 6. The predictions shown demonstrate the model’s ability to localize larger instances of fluids, particularly of the SRF class, with bounding boxes matching ground truth annotations. However, the smaller IRF instances are missed or less precisely localized, consistent with the quantitative results shown previously on this class.

The results of YOLOE on the test partition of the AROI dataset are presented in more detail in Table 3 and are consistent with those of YOLOv12 (Table 2), yet surpassing it on every class. In a similar fashion, the highest performance of YOLOE was achieved on the SRF class with a mAP@50 of 0.847 and a recall of 0.738. PED detection performance was comparable to YOLOv12 at 0.742 mAP@50, indicating robust detection across different biomarkers. The model also showed an increased capacity for IRF detection with a mAP@50 of 0.586 compared to YOLOv12’s 0.553, though this remains the most challenging class for both models.

**Figure 7:**
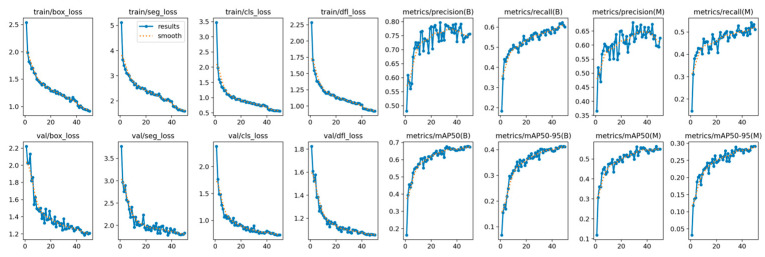
YOLOE training and validation on the AROI dataset showing the model is capable of attaining stability during the training process with minimal overfitting due to the alignment of training and validation.

The training and validation of the YOLOE model can be viewed in Figure 7, illustrating a stable convergence with consistent improvement across all metrics and a good generalization ability with minimal overfitting indicated by the alignment between training and validation.

**Figure 8:**
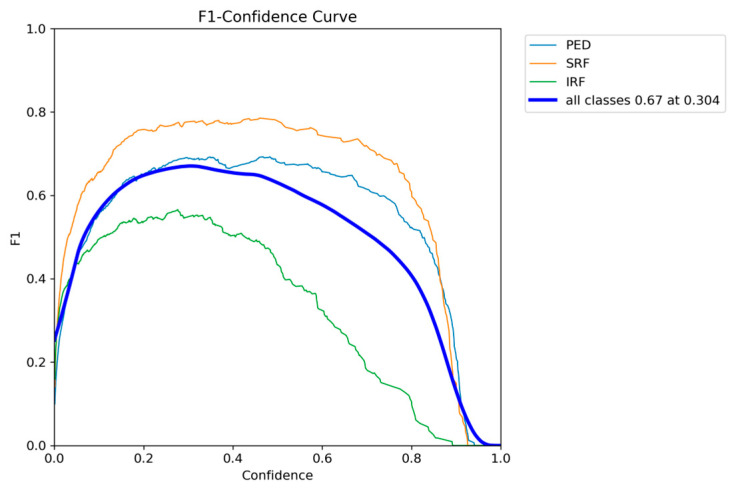
YOLOE F1-confidence curve on the AROI dataset showing the severe drop in performance as the confidence threshold increases for the IRF class.

The F1-confidence curve of YOLOE presented in Figure 8 shows a similar trend to that of YOLOv12, with optimal performance being achieved at a confidence threshold of around 0.3. The F1 score of all classes achieves the highest performance of 0.67 at a threshold of 0.304. Similarly to YOLOv12, SRF and PED maintain their F1 scores on a broad range of confidence thresholds; however, IRF drops with an increase in the threshold value.

**Figure 9:**
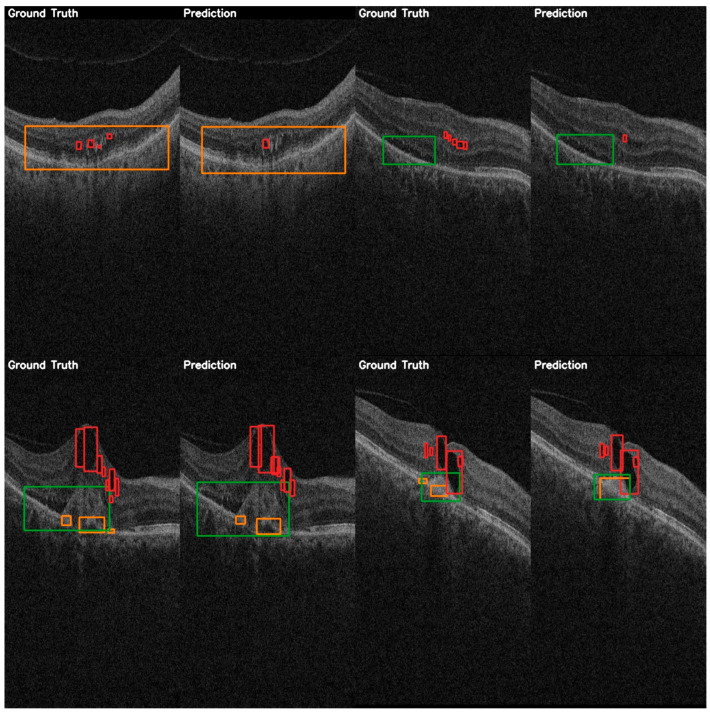
YOLOE validation comparison between ground truth and predictions on the AROI dataset showing the general capability of the model to correctly identify the PED (orange) and SRF (green), while the IRF (red) class is harder to distinguish from the background, and aligning the bounding box is harder to accomplish due to it occupying only small regions.

A comparison between the ground truth annotations and the predicted annotations is shown in Figure 9, and it highlights YOLOE’s detection capabilities. This comparison shows a high localization accuracy even when multiple instances of fluids are present. YOLOE demonstrates a better sensitivity to smaller features, such as IRF, yet there is still a lot of room for improvement, as this class has a significantly lower detection accuracy than the other classes.

### 3.2. Performance Analysis of YOLO Versions on the OCT5k Dataset

The OCT5k dataset [16] presents a more complex picture of pathologies due to the increased diversity of retinal conditions. Overall, the performance of all models was significantly lower compared to that of the AROI dataset, as shown in Table 4, reflecting the inherent complexity of multi-class detection. YOLOv12, which achieved a robust mAP@50 of 0.712 on the AROI dataset, managed to achieve a mAP@50 of only 0.301.

Despite the degradation in performance when comparing the results on the two datasets, the relative ranking of models remained consistent. YOLOE again demonstrates the highest performance across all models with a mAP@50 of 0.355. The results of YOLOE again suggest that its prompt-guided approach may be beneficial for medical applications due to its integration of contextual information. YOLOv12 maintains its position as a second-tier performer with a mAP@50 of 0.301 but is now contested by YOLOv8 and YOLOv11 with 0.301 and 0.304, respectively. RT-DETR continues to underperform significantly when compared to the other models with a mAP@50 of only 0.18, confirming the challenges faced by transformer-based approaches in medical images.

From a computational efficiency perspective, the ranking of models remained stable across datasets, yet they increased across all models, reflecting again the complexity of multiple pathologies. YOLOv12 maintains its position as having the lowest GFLOPs requirement, of only 21.2, and a low inference time, of only 10.4 ms. RT-DETR, again, is the most costly of models from both the perspective of inference time and that of computational cost, while YOLOE continues to have the lowest inference time but a slightly higher computational cost than the other models, except RT-DETR.

The class-wise evaluation of the performance of YOLOv12, shown in Table 5, indicates heavy variation between the detection of different classes, with the highest mAP@50 of 0.722 for geographic atrophy, while fluid, PR layer disruption, and hyperfluorescent spots have not been able to be detected at all. The varying detection accuracy of a subset of classes can be attributed to both small regions and the low number of images and instances in those images. Classes such as soft drusen, which in most cases occupy a larger area, obtain a reasonable detection performance of 0.56 mAP@50, while smaller regions that define hard drusen are more challenging, obtaining a mAP@50 of 0.34. These results align with the known limitation of object detection of smaller objects.

**Figure 10:**
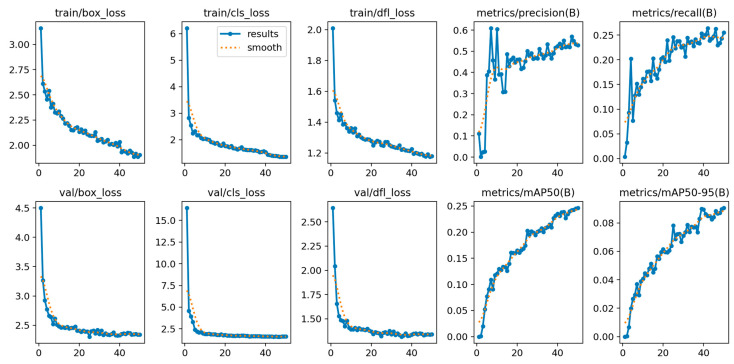
YOLOv12 training and validation on the OCT5k dataset showing the model is capable of attaining stability during the training process with minimal overfitting due to the alignment of training and validation.

The training procedure of YOLOv12, shown in Figure 10, reveals the complexity of the OCT5k dataset, with metrics fluctuating more than on the AROI dataset. However, the model managed to achieve stability, even though the metrics plateaued at lower scores.

**Figure 11:**
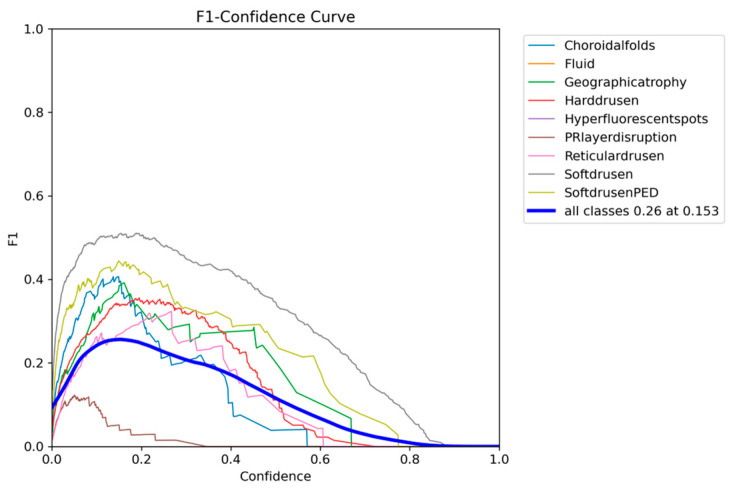
YOLOv12 F1-confidence curve on the OCT5k dataset showing the severe drop in performance as the confidence threshold increases for the PRlayerdisruption class, while the Fluid and Hyperflourecentspots are at 0.

The F1-confidence curve of YOLOv12 presented in Figure 11 shows a consistent drop in F1 as the confidence threshold increases, with an optimum 0.26 F1 being obtained with a lower confidence threshold of 0.153.

**Figure 12:**
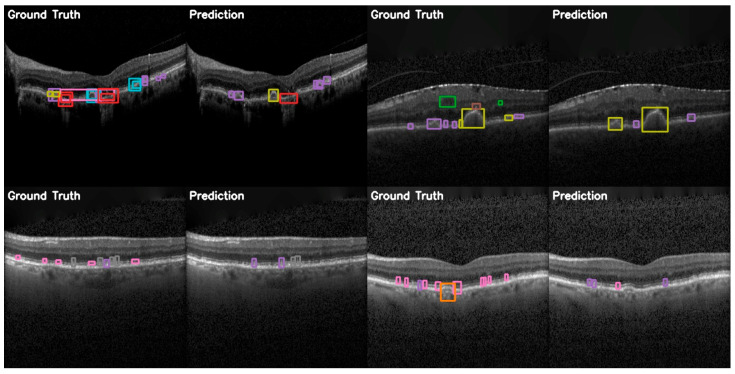
YOLOv12 validation comparison between ground truth and predictions on the OCT5k dataset showing, again, that the model is unable to correctly identify the Fluid class (green) and the Hyperflourescentspots (brown), while the smaller instances of Softdrusen (olive) and Harddrusen (purple) are not found in the predictions of the model.

In the more practical comparative analysis presented in Figure 12, both the successes and limitations of YOLOv12 can be viewed. More prominent pathologies are correctly identified with good localization, while the model struggles in correctly identifying smaller features of certain pathologies and even misses them.

The results of YOLOE on the test partition of the OCT5k datasets are shown in Table 6. YOLOE shows improvements in detecting problematic classes, such as the PR layer disruption, with a mAP@50 of 0.391, whereas YOLOv12 was unable to detect at all. YOLOE outperforms YOLOv12 on all classes except reticular drusen, where it obtains a lower performance of only 0.07 when compared to the mAP@50 of 0.191 of YOLOv12.

**Figure 13:**
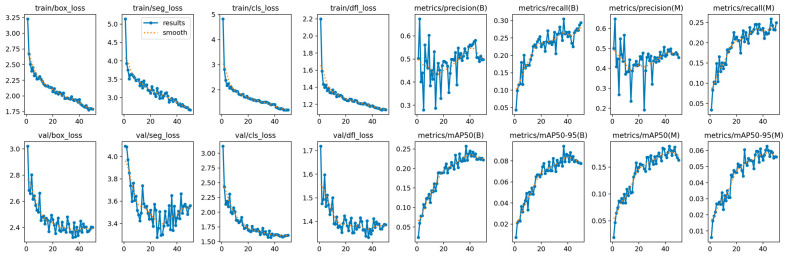
YOLOE training and validation on the OCT5k dataset showing the model is capable of attaining stability during the training process with minimal overfitting due to the alignment of training and validation.

The training of YOLOE shown in Figure 13 indicates a stable convergence with the exception of precision, which oscillates during the training procedure, yet recall shows a steady increase. The alignment between training and validation indicates that the model achieves generalization with minimal overfitting.

**Figure 14:**
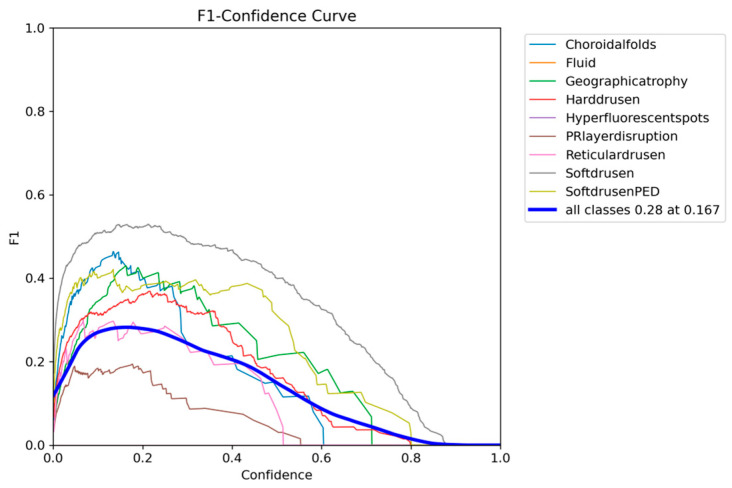
YOLOE F1-confidence curve on the OCT5k dataset showing the severe drop in performance as the confidence threshold increases for the PRlayerdisruption class, while the Fluid and Hyperflourecentspots are at 0.

The F1-confidence curve of YOLOE presented in Figure 14 shows a similar trend to that of YOLOv12, with all classes dropping in F1 as the confidence threshold increases. The optimum F1 score of 0.28 across all classes can be achieved with a confidence threshold of 0.167.

**Figure 15:**
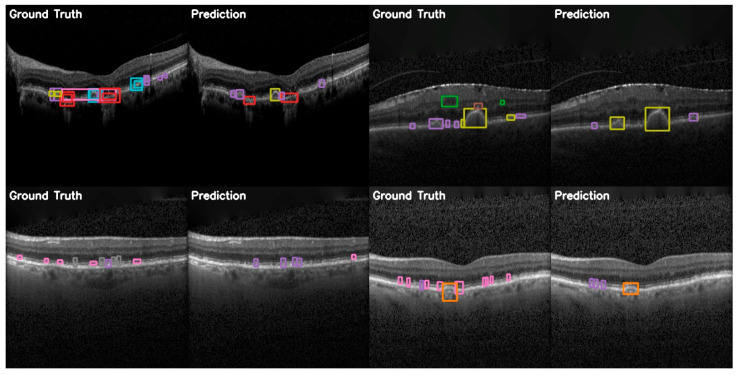
YOLOE validation comparison between ground truth and predictions on the OCT5k dataset, showing again that the model is unable to correctly identify the Fluid class (green) and the Hyperflourescentspots (brown), while the smaller instances of Softdrusen (olive) and Harddrusen (purple) are not found in the predictions of the model.

The comparison between ground truth and predicted annotations shown in Figure 15 demonstrates YOLOE’s superior capacity for detection on the OCT5k dataset. These examples include detections of smaller drusen instances and better localization of classes than the other models. This supports the quantitative analyses presented previously.

## 4. Discussion and Conclusions

Recent studies [7,8,17] have shown that attention-based Unets are capable of performing image segmentation on the AROI dataset with reasonable performance from the perspective of a single metric, specifically the Dice Score. The best results are obtained on the segmentation of layers, which occupy the largest regions in OCT scans, while the performance of fluid segmentation may be improved, likely due to the small regions that they occupy [7,8,17]. The superior performances of YOLOv12 and YOLOE in the AROI dataset [15] can enable clinical applications. For early AMD screenings, the high precision provided by these models in SRF detection (mAP@50 of 0.834–0.847) makes these models a suitable choice as a first-line screening tool by flagging cases requiring the attention of experts. The high precision obtained in the detection of SRF is a valuable finding, as it often indicates a need for treatment as intervention can prevent damage to the retina [37]. Moreover, the consistently high performance in SRF and PED detection (mAP@50 of >0.74) supports a possible application of alarm mechanism in routine screenings for high-volume clinics where immediate expert intervention is not feasible. However, IRF detection remains a challenge due to its small scale, highlighting the need for continued research for the development of better models. Certainly, the accuracy of these models could be improved, especially for the IRF class, through higher image sizes. Nevertheless, this option would increase the computational cost of all models.

OCT5k [16] offers a more robust platform for developing automated retinal image analysis algorithms by providing a larger dataset with multi-disease, multi-grader annotations for both semantic segmentation and object detection tasks, built upon a stringent quality control process. Our evaluation of the OCT5k dataset reveals the true complexity of a general retinal analysis system. Due to its broader area of pathology, the detection accuracy presents a wide variation (ranging from 0% to 72%), indicating that class- or type-specific optimization may be required for a more accurate detection. From a clinical perspective, our work suggests that human–AI collaboration, in a semi-automated setting, may be the most appropriate approach for OCT analysis at the moment. The high detection accuracy of soft drusen (mAP@50 of 0.56–0.60) and geographic atrophy (mAP@50 of 0.71–0.72) allows for automated screening of these pathologies due to their high confidence with minimal human intervention. At the same time, lower confidence classes such as hard drusen (mAP@50 of 0.34) and choroidal folds (mAP@50 of 0.4) could be used in a human–AI collaboration approach where expert verification and confirmation are a requirement.

The reduced computational cost of YOLOv12 (4.9 ms inference time, 21.2 GFLOPs) and YOLOE (4.1 ms inference time) allows for real-time analysis of patients and immediate decision-making. These models may be valuable in rapid triage of suspected cases, automated monitorization of a large number of patients, and remote OCT analysis. However, clinical implementation requires quality assurance protocols, including regular monitoring of the model, validation of edge cases by experts, and clear documentation of diagnoses.

Moving towards a technical perspective, our work provides several findings regarding the application of modern object detection architectures to medical imaging, specifically OCT images for retinal pathology. The performance of YOLOv12 indicates the ability of well-designed attention-based mechanisms to outperform in both accuracy and computational efficiency the heavier transformer-based approaches. The superior performance of YOLOE across both datasets suggests that prompt-guided detection may be a well-suited approach for medical applications due to its ability to incorporate contextual information. YOLOE has been shown to have a strong zero-shot transferability [33], which is a valuable feature for OCT images where pathological features can vary across patients. Its integration of text–visual alignment through its inner mechanisms (LRPC creates internal semantic prompts using its built-in vocabulary, RepRTA refines textual embeddings, enhancing visual–textual alignment) may provide YOLOE with a richer understanding, allowing it to better recognize the smaller features that other models are unable to and classes that appear only in a subset of images.

RT-DETR has been shown to outperform YOLO versions in the literature [38], even on medical image analysis [39]. Specifically for diabetic retinopathy detection, it was shown [39] to outperform YOLOv5 and YOLOv8 in precision, recall, mAP@50, and mAP@50-95. However, in our evaluation, the RT-DETR model significantly underperformed when compared to YOLO variants. This could be attributed to several factors. RT-DETR, being relatively newer, has less domain-specific optimization and pre-training for medical imaging tasks. As a transformer-based model, it requires a larger dataset to achieve stability during training. Whilst YOLO variants have been specifically optimized for small object detection through techniques like Feature Pyramid Networks and anchor optimization, RT-DETR’s global attention mechanism may struggle to focus on these smaller features. RT-DETR’s underperformance indicates that even though transformer-based approaches have demonstrated a high performance in the processing of natural images, their application to medical images may require domain-specific improvements.

Despite its recent introduction, YOLOv12 has already seen various applications with promising results. It has been applied to early detection of sexually transmitted diseases [40], tracking of unmanned aerial vehicles [41], fruit detection [29], and marine litter detection [42], outperforming its predecessors. These results again confirm its ability and suitability for object detection tasks. Our evaluation demonstrates that YOLOv12 and YOLOE represent a significant step forward in retinal OCT analysis through automated detection. These models achieve state-of-the-art performance for fluid detection (through the AROI dataset) while maintaining computational efficiency, allowing for real-time analysis, validating that the results of this study have important implications for clinical practice.

There are several limitations to our work. This study relies on two datasets, which, while well-established, may not fully represent the diversity of OCT imaging conditions encountered. Both datasets exhibit significant imbalance in their classes, with certain pathologies only appearing in a small subset of images. This is highlighted by the inability of the models to correctly identify certain classes. Our analysis of multi-pathology detection (through the OCT dataset) indicates the continued need for domain-specific optimizations and expertise. The models were trained on a standardized image size (640 × 640), which may not be optimal for the detection of small pathologies. While higher images could potentially improve detection (particularly for smaller pathologies such as IRF), there are computational constraints that limit this exploration. The findings presented in our work provide a foundation for the future development of automated OCT analysis systems.

Future research should focus on several areas. One such area is the investigation of few-shot and zero-shot learning approaches to address the cases of rare pathologies where only a limited amount of data can be obtained. The development and investigation of interpretability methodologies is a key requirement for clinical applications. Future research should also prioritize clinical development including validation studies comparing automated detection accuracy against expert diagnoses, cost analyses of automated detection versus the traditional expert review, longitudinal studies to evaluate the impact of automated monitoring instead of the traditional approach, development of explainability methodologies to provide experts with an interpretable reasoning behind the automated process of detection, and the investigation of federated learning approaches to allow for the improvement of the model across multiple sites while maintaining patient privacy.

## Figures and Tables

**Table 1:** Comparative analysis of object detection models on the test partition of the AROI dataset, showing that all models outperform RT-DETR, while YOLOE outperforms all other models and YOLOv12 has the lowest computational cost. Bold values indicate the best performance for each metric.

Metric	YOLOv8	YOLOv9	YOLOv10	YOLOv11	YOLOv12	RT-DETR	YOLOv8-Worldv2	YOLOE
mAP@50	0.691	0.663	0.674	0.694	0.712	0.566	0.697	**0.725**
mAP@50-95	0.462	0.455	0.466	0.473	0.485	0.267	0.444	**0.495**
Mean F1	0.642	0.609	0.629	0.643	0.661	0.613	0.632	**0.675**
Mean Precision	0.789	0.763	0.747	0.797	0.775	0.689	**0.836**	0.786
Mean Recall	0.543	0.514	0.545	0.541	0.581	0.552	0.516	**0.597**
Inference Time [ms]	4.9	6.8	6.3	5.7	4.9	15.3	5.9	**4.1**
Computational Cost [GFLOPs]	28.4	26.7	24.5	21.3	**21.2**	103.4	32.6	35.3

**Table 2:** YOLOv12 results on the test partition of the AROI dataset showing that the IRF classes with the most instances in the fewest classes are the hardest to find due to the fact that they occupy the smallest region.

Class	Images	Instances	Precision	Recall	mAP@50	mAP@50-95
all	107	420	0.762	0.593	0.712	0.485
PED	104	157	0.786	0.631	0.749	0.528
SRF	63	80	0.831	0.738	0.834	0.626
IRF	26	183	0.67	0.41	0.553	0.3

**Table 3:** YOLOE results on the test partition of the AROI dataset showing that the IRF classes with the most instances in the fewest classes are the hardest to find due to the fact that they occupy the smallest region.

Class	Images	Instances	Precision	Recall	mAP@50	mAP@50-95
all	107	420	0.786	0.597	0.725	0.495
PED	104	157	0.764	0.618	0.742	0.541
SRF	63	80	0.868	0.738	0.847	0.642
IRF	26	183	0.727	0.437	0.586	0.302

**Table 4:** Comparative analysis of object detection models on the test partition of the OCT5k dataset, showing that all models outperform RT-DETR, while YOLOE outperforms all other models and YOLOv12 has the lowest computational cost. Bold values indicate the best performance for each metric.

Metric	YOLOv8	YOLOv9	YOLOv10	YOLOv11	YOLOv12	RT-DETR	YOLOv8-Worldv2	YOLOE
mAP@50	0.301	0.282	0.228	0.304	0.301	0.180	0.292	**0.355**
mAP@50-95	0.120	0.102	0.095	0.132	0.111	0.057	0.117	**0.135**
Mean F1	0.278	0.253	0.228	0.268	0.268	0.196	0.245	**0.322**
Mean Precision	0.371	0.340	0.315	0.381	0.382	0.391	0.414	**0.445**
Mean Recall	0.239	0.208	0.191	0.222	0.210	0.270	0.185	**0.269**
Inference Time [ms]	10.6	13.2	13.6	12.5	10.4	25.8	11.5	**10.3**
Computational Cost [GFLOPs]	28.5	26.7	24.5	21.3	**21.2**	103.5	34.0	35.3

**Table 5:** YOLOv12 results on the test partition of the OCT5k dataset showing that certain classes (Hyperfluorescentspots, Fluid) appear in a very low number of images with a low number of instances, resulting in low precision, while the PRlayerdisruption has a similarly low score due to the small regions occupied.

Class	Images	Instances	Precision	Recall	mAP@0.5	mAP@0.5-0.95
All	57	575	0.382	0.21	0.301	0.111
Choroidalfolds	5	10	0.429	0.3	0.397	0.082
Fluid	2	3	0	0	0	0
Geographicatrophy	6	9	0.833	0.556	0.722	0.182
Harddrusen	33	119	0.429	0.252	0.339	0.112
Hyperfluorescentspots	5	8	0	0	0	0
PRlayerdisruption	23	101	0	0	0	0
Reticulardrusen	9	33	0.312	0.152	0.191	0.0672
Softdrusen	38	265	0.739	0.374	0.56	0.279
SoftdrusenPED	9	27	0.7	0.259	0.503	0.279

**Table 6:** YOLOE results on the test partition of the OCT5k dataset showing that certain classes (Hyperfluorescentspots, Fluid) appear in a very low number of images with a low number of instances, resulting in low precision, while the PRlayerdisruption has a similarly low score due to the small regions occupied, however slightly higher than that of other models.

Class	Images	Instances	Precision	Recall	mAP@0.5	mAP@0.5-0.95
All	57	575	0.445	0.269	0.355	0.135
Choroidalfolds	5	10	0.571	0.4	0.501	0.132
Fluid	2	3	0	0	0	0
Geographicatrophy	6	9	0.75	0.667	0.713	0.236
Harddrusen	33	119	0.468	0.37	0.402	0.139
Hyperfluorescentspots	5	8	0	0	0	0
PRlayerdisruption	23	101	0.65	0.129	0.391	0.111
Reticulardrusen	9	33	0.143	0.0303	0.0744	0.0372
Softdrusen	38	265	0.752	0.457	0.599	0.29
SoftdrusenPED	9	27	0.667	0.37	0.517	0.266

## Data Availability

The data used in this study are openly available: AROI: https://ipg.fer.hr/ipg/resources/oct_image_database (upon request from the authors), OCT5k: https://rdr.ucl.ac.uk/articles/dataset/OCT5k_A_dataset_of_multi-disease_and_multi-graded_annotations_for_retinal_layers/22128671 (accessed on 29 June 2025).

## Abbreviations

The following abbreviations are used in this manuscript:

mAP	Mean Average Precision
mAP@50	Mean Average Precision at an IoU threshold above 0.5
mAP@50-95	Mean Average Precision at IoU thresholds from 0.5 to 0.95
IoU	Intersection over Union
OCT	Optical Coherence Tomography
PED	Pigment Epithelial Detachment
SRF	Subretinal Fluid
IRF	Intraretinal Fluid
